# Over-ground walking or robot-assisted gait training in people with .multiple sclerosis: does the effect depend on baseline walking speed and disease related disabilities? A systematic review and meta-regression

**DOI:** 10.1186/s12883-019-1321-7

**Published:** 2019-05-08

**Authors:** Martin Sattelmayer, Odile Chevalley, Ruedi Steuri, Roger Hilfiker

**Affiliations:** University of Applied Sciences and Arts Western Switzerland Valais (HES-SO Valais-Wallis), School of Health Sciences, Leukerbad, Switzerland

**Keywords:** Robot-assisted gait training, Multiple sclerosis, Over-ground walking, Meta-regression, systematic review

## Abstract

**Background:**

It was suggested that robot-assisted gait training (RAGT) should not be routinely provided to disabled patients in place of conventional over-ground walking training (CGT). There exist several randomised controlled trials reporting on RAGT for people with multiple sclerosis. However, the effectiveness of RAGT varies between studies with the effectiveness pointing in different directions. It might be possible that the effectiveness of RAGT and CGT depends on the disease related disabilities of the people included in the clinical studies. We aimed to systematically search RCTs and to perform a meta-regression to compare the effects of robot-assisted gait training in people with less and higher disease related disabilities. The Expanded Disability Status Scale (EDSS) scores were used to classify level of disability.

**Methods:**

A systematic search was developed to search four electronic databases (MEDLINE, CENTRAL, EMBASE and CINAHL) for eligible articles. A random effects model was applied to meta-analyse the effects of the interventions. Meta-regression was performed with an uni-variable random effects model using baseline walking speed and EDSS to predict the between group effect.

**Results:**

The search on databases resulted in 596 records and finally nine studies were included into the review. The pooled estimates of the effects for performance over short and long distance tests were small and non-significant: -0.08 SMD (95% CI: -0.51 to 0.35) and − 0.24 SMD (95% CI: -0.67 to 0.19). Neither baseline walking speed or disease related disability were related to the mean effect size.

**Discussion:**

Future studies are needed to help clinicians to decide, which intervention should be allocated to the individual patient.

**Electronic supplementary material:**

The online version of this article (10.1186/s12883-019-1321-7) contains supplementary material, which is available to authorized users.

## Background

Mobility impairments and especially gait impairments have been described as a frequently occurring consequence of Multiple Sclerosis (MS) [1]. Gait abnormalities are reported with high prevalence in MS and might affect the quality of life of people with MS [2]. Furthermore, assistive devices are required to maintain mobility in later stages of the disease [3]. Comber and colleagues [4] evaluated in a meta-analysis of 41 studies that a variety of gait abnormalities occur in people with MS. Among others a large effect was observed on stride length, velocity, double support duration, step length and swing phase duration.

Goldman and co-workers [5] reported that changes in walking distance are associated with the level of disability. A reduced walking distance was already observed in people with mild disabilities in comparison to healthy controls. It has been found that the walking distance decreases continuously, with the shortest distance occurring in people with severe disabilities. Furthermore, gait abnormalities are reported even in people with minimal neurological signs. Martin et al. [6] compared in a three-arm observational study gait parameters of people with recently diagnosed MS and less impairments on the Expanded Disability Status Scale (EDSS) with the gait parameters of a control group. People with MS and signs of pyramidal tract lesions showed subtle changes regarding stride length, gait speed and an elongated double limb support phase. These changes were also observed in a MS subgroup with no pyramidal signs, which indicates that gait problems exist already in early stages of MS even in people without clear pyramidal tract signs. Next to altered kinematic parameters the investigators also identified a changed pattern of ankle muscle activation in people with early stage MS. However, gait changes were only detected with the help of laboratory measures. Clinical investigations and observational assessments are probably not sensitive enough to detect such subtle gait abnormalities [6]. Altered gait parameters in people with MS may have different causes. Pyramidal tract lesions, proprioceptive deficits and cerebellar lesions have all been described as causing gait disturbances [7]. They all might reduce the maximal walking distance in people with MS, which is frequently impaired in people with MS [8].

Disease related disability is often classified with the framework of the EDSS scale. The scale ranges from 0 to 10 points (0: normal neurological examination and 10: death due to MS). Walking ability is a major element within this framework. For example, to achieve a score of 6.0 on the EDSS, assistance is required to walk about 100 m (i.e. assistance may mean resting, the use of unilateral aids at most times, or the intermittent use of bilateral aids. The assistance of another person also counts as “with aid”) [9]. Disease related mobility is broadly classified for people with moderate and high impairments. Less information is used to classify mobility deficits of people with a lower degree of functional impairment [7].

### Description of the intervention

One intervention widely used in clinical practice to improve gait problems is robot-assisted gait training (RAGT). Robot-assisted gait training is a training in which the patient’s body weight is supported and the gait movement is assisted. Morone and colleagues [10] reported the following definition and categories of RAGT devices: i) the devices are capable of mobility with different levels of autonomy, ii) they can be classified as either “exoskeletons” (i.e. the movement of specific joints is controlled (such as hip, knee or ankle joint)) or “end-effect robots” (the device is at the end of the leg, i.e. the feet are placed on a footplate) and iii) the devices can be classified as static (i.e. the patient remains in a fixed environment) or dynamic (i.e. capable to change the location).

The first randomized controlled trial evaluating robot-assisted gait training in people with MS was published by Beer et al. [11]. The study appraised RAGT as promising intervention with moderate to large effects on gait speed in people with MS. However, other studies showed less clear results [12, 13]. Therefore, it was suggested that RAGT should not be routinely provided to disabled patients in place of conventional over-ground walking training (CGT) outside of controlled clinical studies [14].

### How and why the intervention might affect subgroups with different disease related disabilities differently

If a person with MS can walk over-ground without weight support and without a certain speed, the training on a robot-assisted gait trainer is probably less challenging than conventional over-ground walking training, which requires the control of more degrees of freedom. In addition, the individual must perform constant balance reactions to ensure postural control. Furthermore, the robotic systems used in the first published studies had a maximal speed limit (e.g. limited to a possible maximal speed of 3.2 km per hour (i.e. max. 0.89 m per second)). This speed might be too slow for patients with better walking abilities and a higher walking speed would be needed for challenging training conditions. Therefore, conventional over-ground gait rehabilitation interventions not using robotic assistance might present a more challenging training for people with higher abilities.

To know whether robot-assisted gait training is better-suited for slow walkers than for faster walkers is important for the optimal use of scarce resources (i.e. device use). The best study to answer this question would be an RCT with subgroups (i.e. to compare a higher walking ability group with a lower walking ability group) or a regression within an RCT with walking ability as a co-variable. However, such an RCT would need a large number of participants in each subgroup and would consequently be associated with considerable resources. Therefore, the existing data should be used to try to answer such a question with a standard meta-analysis with a meta-regression. If results of such a meta-regression are promising, a large multicentre RCT could be performed.

### Objectives

The objectives of this study were to evaluate i) whether a robot assisted gait training or conventional over-ground walking training was more effective on the outcomes walking performance over short or long distance walking tests; ii) whether studies in which patients had a lower baseline walking speed at baseline showed a larger between group (i.e. robot-assisted walking versus conventional over-ground walking) difference compared to studies with patients with a higher baseline walking speed at baseline and iii) studies in which patients had a higher disease related mobility impairment (i.e. higher EDSS score) at baseline showed a larger between group difference compared to studies with patients with a lower disease related mobility impairment (i.e. lower EDSS score) at baseline.

## Methods

During the whole review process the PRISMA statement [15] was followed to increase clarity of reporting. A protocol of this review was written but not published a priori.

### Criteria for considering studies for this review

- Only studies reporting about adult people with multiple sclerosis were eligible.
- Studies were eligible when they compared a robot-assisted gait training with another (non-robot-assisted) gait training.
- Studies had to report about at least one of the pre-specified outcomes of interest: walking performance over short (measured with tests such as the 10 Metre Walk Test) or walking performance over longer distances (measured with tests such as the 6 Minute Walk Test).
- Only randomised controlled studies were included.
- No language restrictions were set to include studies.

### Search methods for identification of studies

The following databases were searched from inception to September 23th 2016 by MS and RH: Medline (via PubMed), the Cochrane Central Register of Controlled Trials, EMBASE and CINAHL with a combination of keywords related to robot-assisted gait training and multiple sclerosis (see Additional file 1 for full search strategy). In addition, Google Scholar was searched for articles citing Beer et al. [11]. Furthermore, the reference lists of the included articles were searched for eligible studies. Specifically, four clinical trial registers were investigated to identify additional studies (https://clinicaltrials.gov; https://www.clinicaltrialsregister.eu/; http://www.controlled-trials.com/; http://www.umin.ac.jp/ctr/).

All retrieved records were imported in an electronic literature management system. First, duplicates were removed electronically, then two reviewers (MS, RH) independently screened titles and abstracts of the records. Afterwards, the full-texts of the remaining records were read by the two reviewers (MS, RH) and included in the systematic review when they fulfilled all inclusion criteria. In case of disagreement, a third reviewer was involved to decide about study selection (OC).

### Risk of bias assessment

The Cochrane Risk of Bias tool [16] was used to evaluate the risk of bias of the included studies. Two reviewers (MS, OC) independently assessed the random sequence generation, allocation concealment, blinding of participants and personnel, blinding of outcome assessment, incomplete outcome data, selective reporting and other bias. Evaluation of the small study effect (which could indicate publication bias): Because tests for funnel plot asymmetry (i.e. indication for a small study effect) should only be done when there are at least ten studies [17], we did not statistically test for this. However, we plotted funnel plots and inspected them visually for asymmetry.

### Data collection

The primary outcome measure of this review was walking performance over short distances (e.g. measured with the 10 Metre Walk Test or assessments using a similar distance). The secondary outcome measure was walking performance over long distances, which could be evaluated with measurement instruments such as the 6 Minute Walk Test. Both outcome measurements provide different information about the concept “walking ability” in people with MS. Kieseier and Pozzilli [18] reported in their systematic review that short walking tests represent good measures of the overall walking ability and longer distance tests provide information about walking fatigability and maximal walking distance limitations.

For the meta-regression, we extracted the walking speed and the EDSS evaluations at baseline to estimate the disease related mobility impairment (i.e. the EDSS score were treated as continuous variable). For the walking speed, we extracted the speed for short walking distances, if available, otherwise the speed for long distances.

All data were extracted by two reviewers (MS, RH). One reviewer extracted the data and another controlled the data. Means and standard deviation were extracted for change from baseline values to values immediately after the intervention [11, 12, 19–22]. If change values were not available final scores at the first assessment after the intervention were used [13, 23]. For crossover trials [12] we used data from the first period only, because of potential carry-over effect of the gait training.

### Statistical analysis

Standardized mean differences (SMD) (Hedges’ *g* (adjusted for small sample sizes)) were calculated by dividing the between group differences of the means by the pooled standard deviation of the outcomes. Effects were weighted with an inverse of the variance. An effect size of 0.2 was considered as small, one of 0.5 as moderate and of 0.8 as large effect [24]. A positive standardised mean effect size indicated a larger change in the CGT and a negative effect size indicated a larger change in the RAGT group. The meta-analysis was performed using a random-effects model. Between trial heterogeneity was quantified using I^2^ statistics (i.e. I^2^ values of 25% indicate low, 50% moderate and 75% high heterogeneity [25]). The meta-analysis was performed in Review Manager 5.3.

Two moderator variables were explored with meta-regression. First, we hypothesized that participants with a lower baseline walking speed would benefit more from RAGT compared to participants with higher speed.

Second, we hypothesized that patients with higher disease related disabilities (i.e. higher EDSS scores) will benefit more from the robot-assisted gait training compared to patients with lower disabilities (i.e. lower EDSS scores). To evaluate both hypotheses, uni-variable random effects meta-regressions with the between group difference (standardized mean difference) as dependent variable and the i) baseline walking speed or ii) baseline EDSS score as independent variables were performed (i.e. the EDSS score was treated as continuous variable). The meta-regressions was performed in Stata 14.1 with the metareg command [26]. The EDSS score and the baseline walking speed were used as moderator variable for the between group difference. Therefore, they were used in both groups.

## Results

### Results of the search

**Fig. 1:**
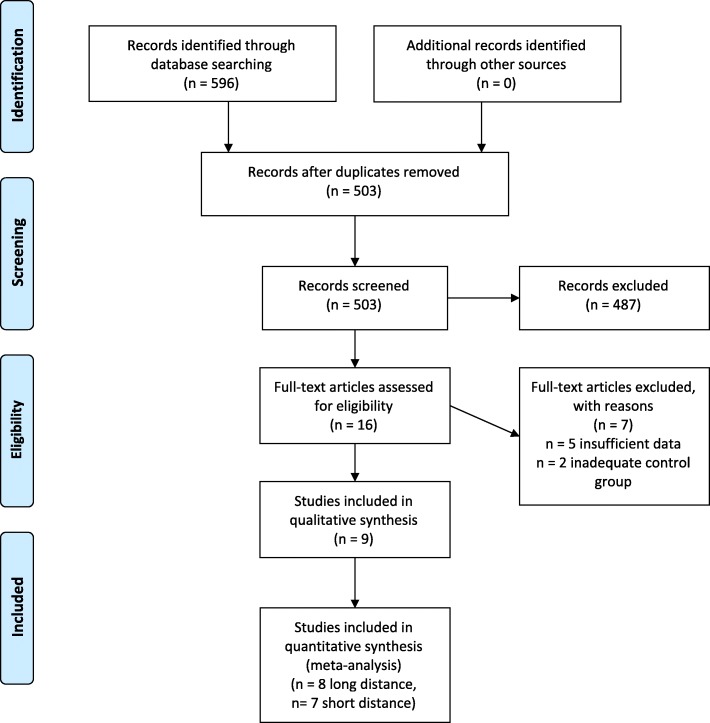
Study flow

The search on databases resulted in 596 records. After deletion of duplicates 503 records remained. Screening of titles and abstracts reduced the number of eligible records to 16, which were screened as full text articles. During this stage seven articles were removed. Five articles were conference proceedings and contained insufficient information for analysis [27–31]. Two studies were excluded because of an inadequate comparison group (i.e. the comparison group did not perform a conventional gait training) [32, 33]. Finally, nine studies were included in this systematic review [11–13, 19–23, 34]. Seven studies were included for the analysis of the short distance walking tests [11–13, 19–22] and eight studies were included into the analyses of the long distance walking tests [11–13, 19–23]. The study flow is presented in Fig. 1.

### Included studies

The nine included studies reported about 309 participants. The average EDSS score ranged between 5.0 [12] and 6.62 [23]. Concordantly, the fastest average walking speed at baseline was reported by Lo and Triche [12] (0.78 m/s) and the slowest average walking speed occurred in the study of Beer and colleagues [11] (0.23 m/s). The majority of studies used the Lokomat for the robot-assisted gait training and only one study [23] used the gait trainer GTII for the robot assisted training. The CGT intervention varied between studies. Some studies included training modalities such as stretching and strengthening [24], balance and co-ordination exercises [23] or sit to stand exercises into their group intervention. However, all studies used an active comparator (i.e. robot-assisted gait training was not compared against no intervention) and the volume of the training (time spent training) was similar during intervention and control groups in all studies. None of the included studies reported serious adverse events that were caused by either the RAGT or CGT intervention. Minor issues were reported by Beer et al. [11] (i.e. skin irritations after RAGT were observed in two participants).

Key characteristics of the included studies are presented in Table 1.

**Table 1:** Characteristics of included studies

Study	Country	N randomised (RAGT/CGT)	N post-treatment (RAGT/CGT)	Mean age (RAGT/CGT)	EDSS (RAGT/CGT)	Mean walking speed at baseline (m/s) (RAGT/CGT)	Follow up points	Procedures in RAGT group	Procedures in CGT group	Primary outcome measure	Secondary outcome measure
Beer et al. [11]	Switzerland	35 (19/16)	29 (14/15)	49.7 (SD: 11.0)/51.0 (SD: 15.5)	6.5 (r: 6–7.5)/6.5 (r: 6–7.5)	0.21 (IQR: 0.09–0.27)/0.24 (IQR: 0.17–0.49)	6 months	RAGT (Lokomat); 15 sessions (30 min) over 3 weeks, maximal speed: 2.8 km/h	Conventional walking training; 30 min training with assistance of a physiotherapist, 15 sessions over 3 weeks	20MWT (walking velocity)	6MWT (walking distance), stride length, knee extensor strength, EBI (ADL independence), VAS (walking safety and satisfaction)
Lo and Triche [12]	US	13 (6/7)	13 (6/7)	50.2 (SD: 11.4)/49.8 (SD: 11.8)	5.0 (SD: 1.6)/4.9 (SD: 0.9)	0.87 (SD: 0.31) 0.70 (SD 0.32)	No follow up	RAGT (Lokomat): 6 sessions (40 min) over 3 weeks, maximal speed 2.5 km/h	BWSTT; 6 sessions (40 min) over 3 weeks, maximal speed 2.5 km/h	T25FW (walking velocity)	6MWT (walking distance), double support time and step length ratio (spatiotemporal gait kinematics)
Giesser et al. [22]	US	36 (18/18)	36 (18/18)	n.a.	6.5/6.0	n.a.	No follow up	RAGT (Lokomat): 3 sessions (30–40 min) per week over 12–16 weeks	Resistance training with elastic bands and weights; 3 sessions (30–40 min) per week over 12–16 weeks	T25FW (walking velocity)	6MWT (walking distance), fatigue, depression
Wier et al. [34]	as reported in [12]									MS Quality of Life Inventory	FSS (fatigue), Life Satisfaction
Vaney et al. [19]	Switzerland	67 (34/33)	49 (26/23)	58.23 (SD: 9.42)/54.22 (SD: 11.28)	5.88 (SD: 0.9)/5.72 (SD: 1.06)	0.52 (SD: 0.32)/0.6 (SD: 0.34)	9 months	RAGT (Lokomat): 9 sessions of 30 min	Walking in a group with a physiotherapist (inside and outside); 9 sessions of 30 min	Well-Being VAS (QoL), EQ-5D (QoL), accelerometer (activity level), 10MWT (walking speed), 3MWT (walking distance)	BBS (balance), Würzburger Erschöpfungsinventar bei MS (fatigue), RMI (mobility), modified Ashworth Scale (spasticity), NRS (pain)
Schwartz et al. [13]	Israel	32 (15/17)	28 (12/16)	46.8 (SD: 11.5)/50.5 (SD: 11.5)	6.2 (SD: 0.5)/6 (SD: 0.6)	0.49 (SD: 0.3)/0.53 (SD: 0.31)	3 and 6 months	RAGT (Lokomat): 2–3 sessions (30 min) per week for 4 weeks, maximal speed: 3 km/h	Gait, dynamic balance and standing from sitting exercises, 2–3 sessions (30 min) per week for 4 weeks	10MWT (walking speed), 6MWT (walking distance), TUG (mobility)	BBS (balance), FIM (daily living functions), RAND-36 (health-related QoL)
Straudi et al. [21]	Italy	18 (9/9)	16 (8/8)	49.6 (SD: 12.0)/61.0 (SD: 8.8)	5.8 (SD: 0.8)/5.7 (SD: 0.7)	0.55 (SD: 0.24)/0.46 (SD: 0.22)	3 months	RAGT (Lokomat): 12 sessions (30 min) over 6 weeks, maximal speed: 3 km/h	Conventional therapy: Stretching and strengthening of lower limb muscles, optional motor coordination and balance exercises were integrated), 12 sessions (1 h) over 6 weeks	6MWT (walking distance), TUG (mobility)	FSS (fatigue), spatiotemporal gait parameters (gait speed, cadence, double support, step length and time), pelvis and hip kinematic profiles
Straudi et al. [20]	Italy	58 (30/28)	55 (27/28)	52.26 (SD: 11.1)/54.12 (SD: 11.4)	6.43 (SD: 0.38)/6.46 (SD: 0.43)	0.59 (SD: 0.37)/0.45 (SD: 0.24)	3 months	RAGT (Lokomat): 12 sessions (1 h) over 6 weeks, maximal speed: 3 km/h	Conventional therapy: Stretching and strengthening of lower limb muscles, motor coordination and gait and balance exercises,å 12 sessions (1 h) over 6 weeks	10MWT (walking speed), 6MWT (walking distance)	BBS (balance), TUG (mobility), FSS (fatigue), PHQ-9 (depression), SF-36 (health-related QoL), VAS (treatment acceptance)
Pompa et al. [23]	Italy	50 (25/25)	43 (21/22)	47.00 (SD: 11.2)/49.86 (SD: 8.21)	6.62 (SD: 0.42)/6.50 (SD: 0.49)	0.28 (SD: 0.13) /0.34 (SD: 0.19)	n.a.	RAGT (Gait Trainer GT II): 3 sessions (40 min) a week over 4 weeks, maximal speed: 1.8 km/h	Conventional walking therapy: Preparation exercises within parallel bars, trunk and pelvis control exercises, balance and coordination exercises and walking exercises, 3 sessions (40 min) a week over 4 weeks	2MWT (walking distance), FAC (ambulation)	RMI (global mobility), mBI (independence in ADL), EDSS (MS severity), FSS (fatigue), VAS (spasticity)

*20MWT* 20 Metre Timed Walking Test, *10MWT* 10 Metre Walk Test, *6MWT* 6 Minute Walk Test, *3MWT* 3 Minute Walk Test, *2MWT* 2 Minute Walk Test, *ADL* Activities of daily living, *BWSTT* Body weight supported treadmill training, *CGT* Conventional over-ground walking training, *EBI* Extended Barthel Index, *EQ-5D* EuroQol-5D, *FAC* Functional Ambulation Categories, *FIM* Functional Independence Measure, *IQR* Interquartile range, *mBI* Modified Barthel Index, *NRS* Numeric rating scale, *QoL* Quality of Life, *RAGT* that robot-assisted gait training, *r* Range, *RMI* Rivermead Mobility Index, *SD* Standard deviation, *SF-36 (or RAND-36)* Short Form (36) Health Survey, *T25FW* Timed 25-ft walk, *VAS* Visual analogue scale

### Findings

#### Meta-analysis walking performance over short distances

For the primary outcome walking performance over short distances seven studies reporting about 224 people with MS could be used for the meta-analysis. Per study one effect was included into the meta-analysis. The outcome measures used varied between studies. Three studies administered the 10 Metre Walk Test [13, 19, 20], Beer and colleagues [11] used the 20 Metre Walk Test, the timed 25 Foot Walk Test was applied in two studies [12, 22] and Straudi et al. [21] used laboratory measures to evaluate the walking speed. With exception of Schwartz et al. [13] it was possible to extract change from baseline values for the analysis.

**Fig. 2:**
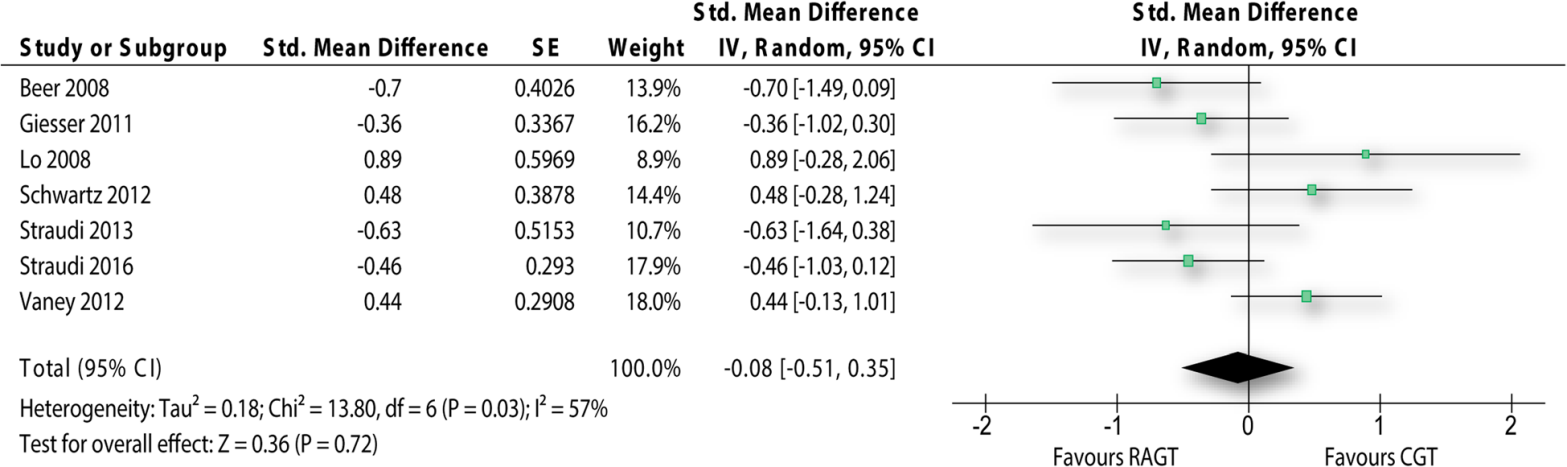
Forest plot walking performance over short distances. RAGT: robot-assisted gait training; CGT: conventional over-ground walking

The pooled effect size of the comparison was − 0.08 SMD in favour of robot-assisted gait training with a 95%CI between − 0.51 and 0.35 (Fig. 2). However, the effect was very small and statistically not significant (p: 0.72). Statistical heterogeneity was between moderate and high (I^2^: 57%).

#### Meta-regression baseline walking speed as predictor for walking performance over short distances

**Fig. 3:**
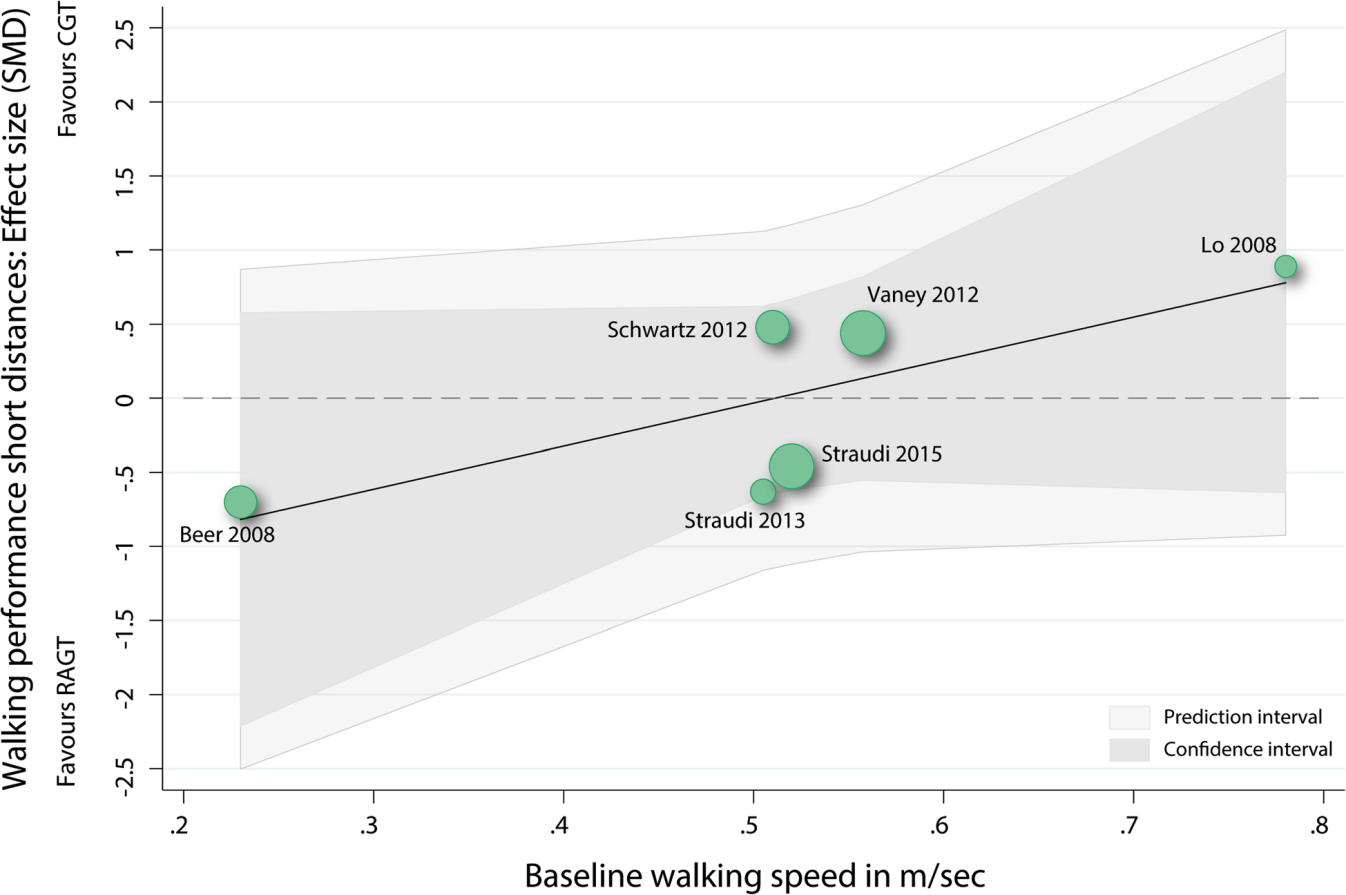
Scatterplot meta-regression with baseline walking speed as predictor for walking performances over short distances. RAGT: robot-assisted gait training; CGT: conventional over-ground walking

A univariable meta-regression was performed to analyse whether the walking speed at baseline was an independent predictor of the performance over short distance walking tests after the intervention phase. Neither the overall model (p: 0.11, r2: 0.65) or the baseline walking speed variable (b1: 0.29 (95%CI: -0.11 to 0.69), t: 2.03, p: 0.11) were significantly related to the mean effect size (Fig. 3).

#### Meta-regression baseline EDSS score as predictor for walking over short distances

**Fig. 4:**
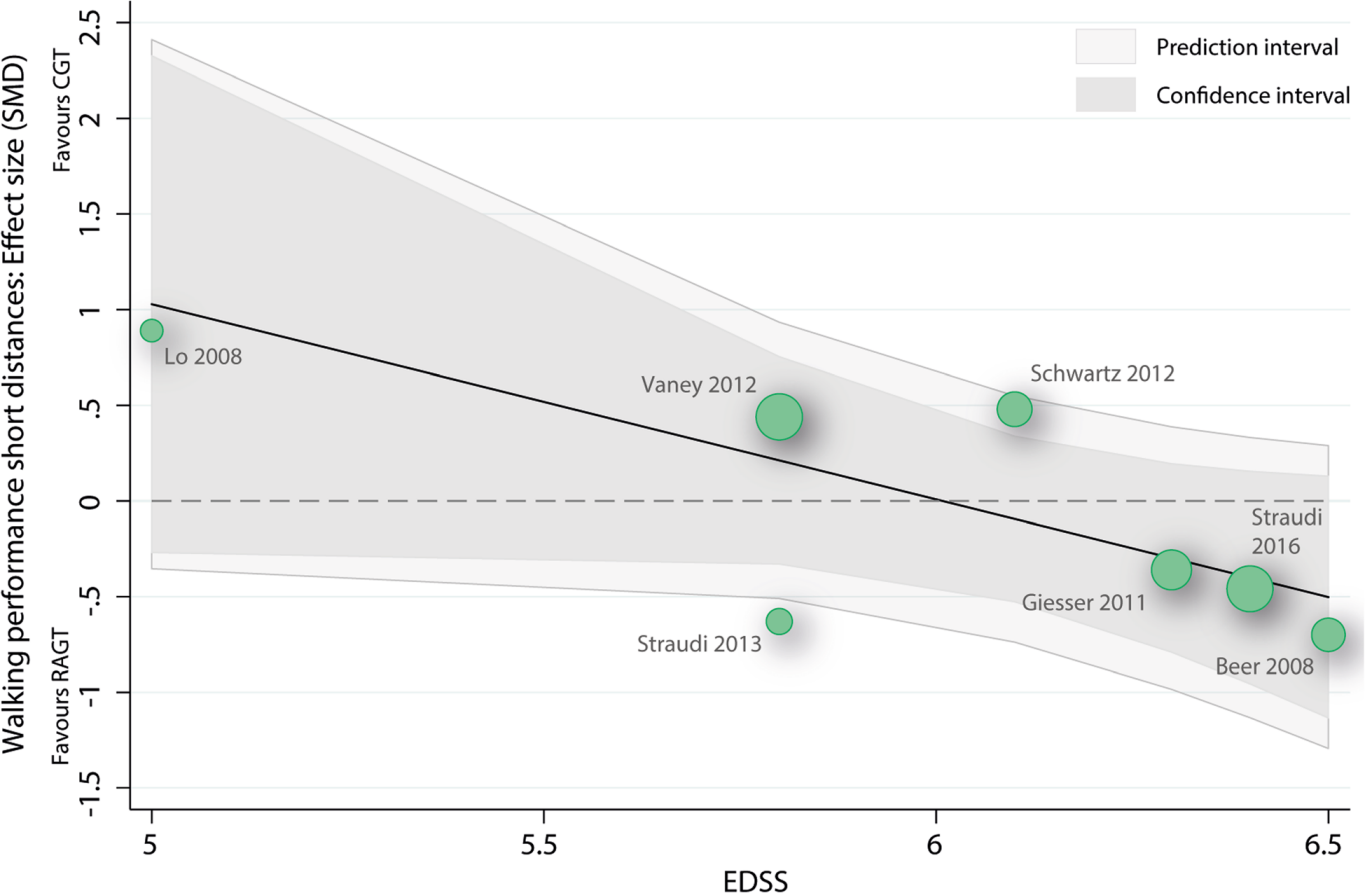
Scatterplot meta-regression baseline EDSS score as predictor for walking performances over short distances. RAGT: robot-assisted gait training; CGT: conventional over-ground walking; EDSS: Expanded Disability Status Scale

To analyse whether the initial EDSS score within studies had an influence on the outcome walking speed an uni-variable meta-regression was performed with walking speed as dependent variable and EDSS as independent variable. Neither the overall model (p: 0.053, r2: 0.85) or the baseline EDSS variable (b1: -1.02 (95%CI: -2.06 to 0.02), t: − 2.52, p: 0.053) were significantly related to the mean effect size (Fig. 4).

#### Meta-analysis walking performance over long distances

**Fig. 5:**
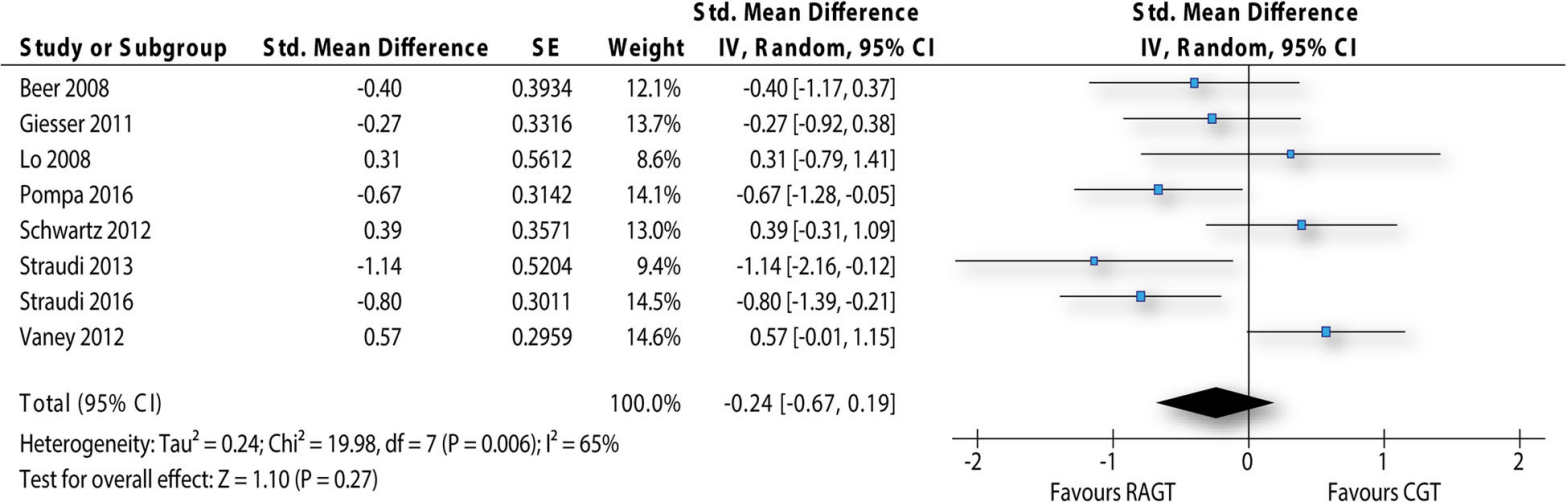
Forest plot walking performance over long distances. RAGT: robot-assisted gait training; CGT: conventional over-ground walking

Eight studies were included into the comparison robot-assisted gait training against conventional over-ground walking for the outcome walking performance over long distances (Fig. 5). Per study one effect was included into the meta-analysis. In total 272 participants were included in this analysis. Studies administered three different outcome measures within this analysis. Most studies used the 6 Minute Walk Test, only Pompa et al. [23] and Vaney and co-workers [31] used walking tests with shorter time periods (2 and 3 min respectively). Change values for the walking performance between baseline and first follow up assessment could be used for the analysis. Only Schwartz et al. [13] reported final values at the first follow up assessment.

The pooled estimate of the effect was − 0.24 SMD (95% CI: -0.67 to 0.19) and showed a small not significant effect in favour of robot-assisted gait training (p: 0.27). Statistical heterogeneity within this analysis was between moderate and high (I^2^: 65%).

#### Meta-regression baseline walking speed as predictor for walking performance over long distances

**Fig. 6:**
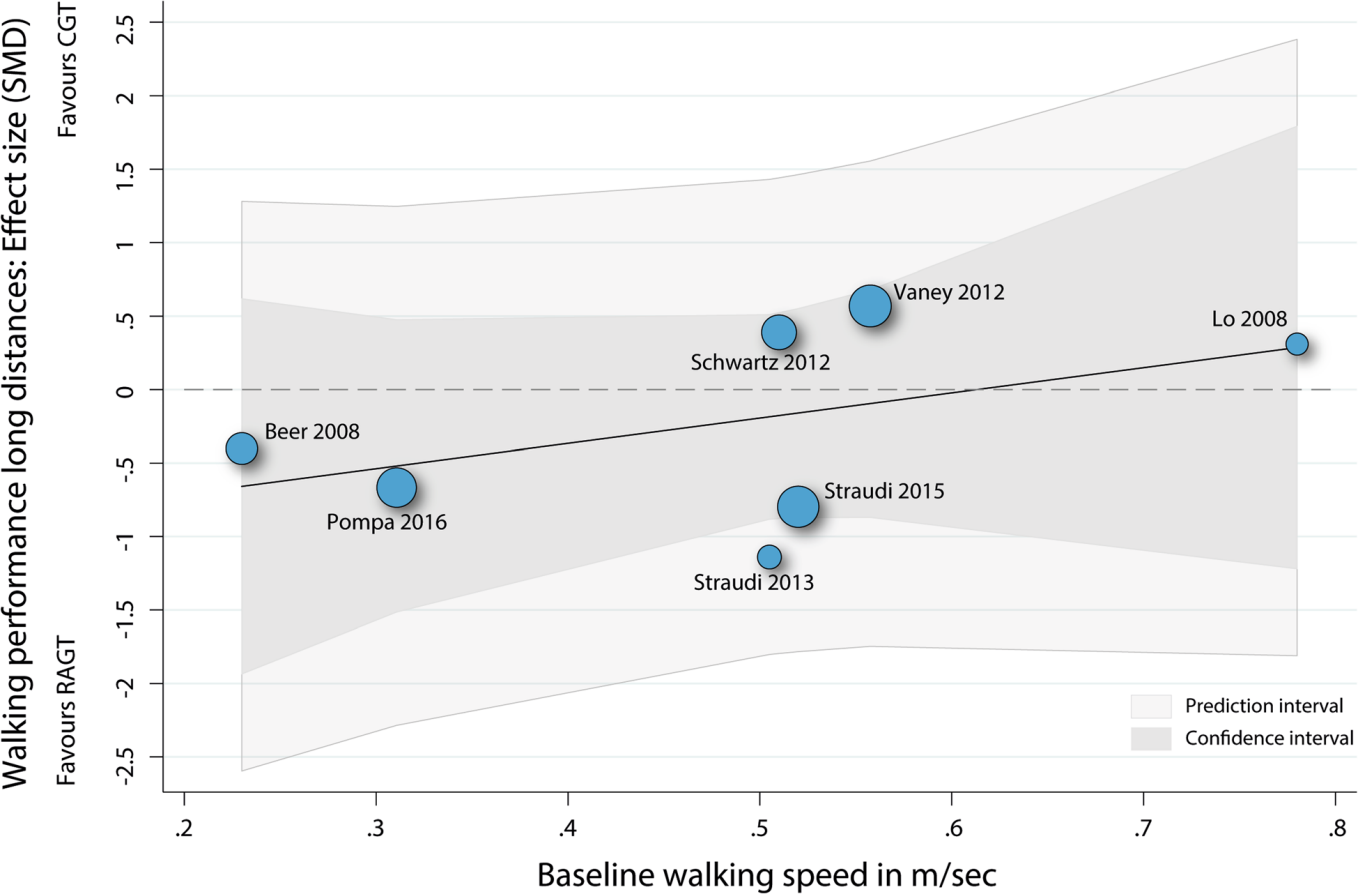
Scatterplot meta-regression baseline walking speed as predictor for walking performances over long distances. RAGT: robot-assisted gait training; CGT: conventional over-ground walking

A univariable meta-regression was performed to analyse whether the walking speed at baseline was an independent predictor of the performance over long distance walking tests after the intervention phase (Fig. 6). Neither the overall model (p: 0.33, r2: 0.04) or the baseline walking speed variable (b1: 0.17 (95%CI: -0.24 to 0.58), t: 1.08, p: 0.33) were significantly related to the mean effect size.

#### Meta-regression baseline EDSS score as predictor for walking over long distances

**Fig. 7:**
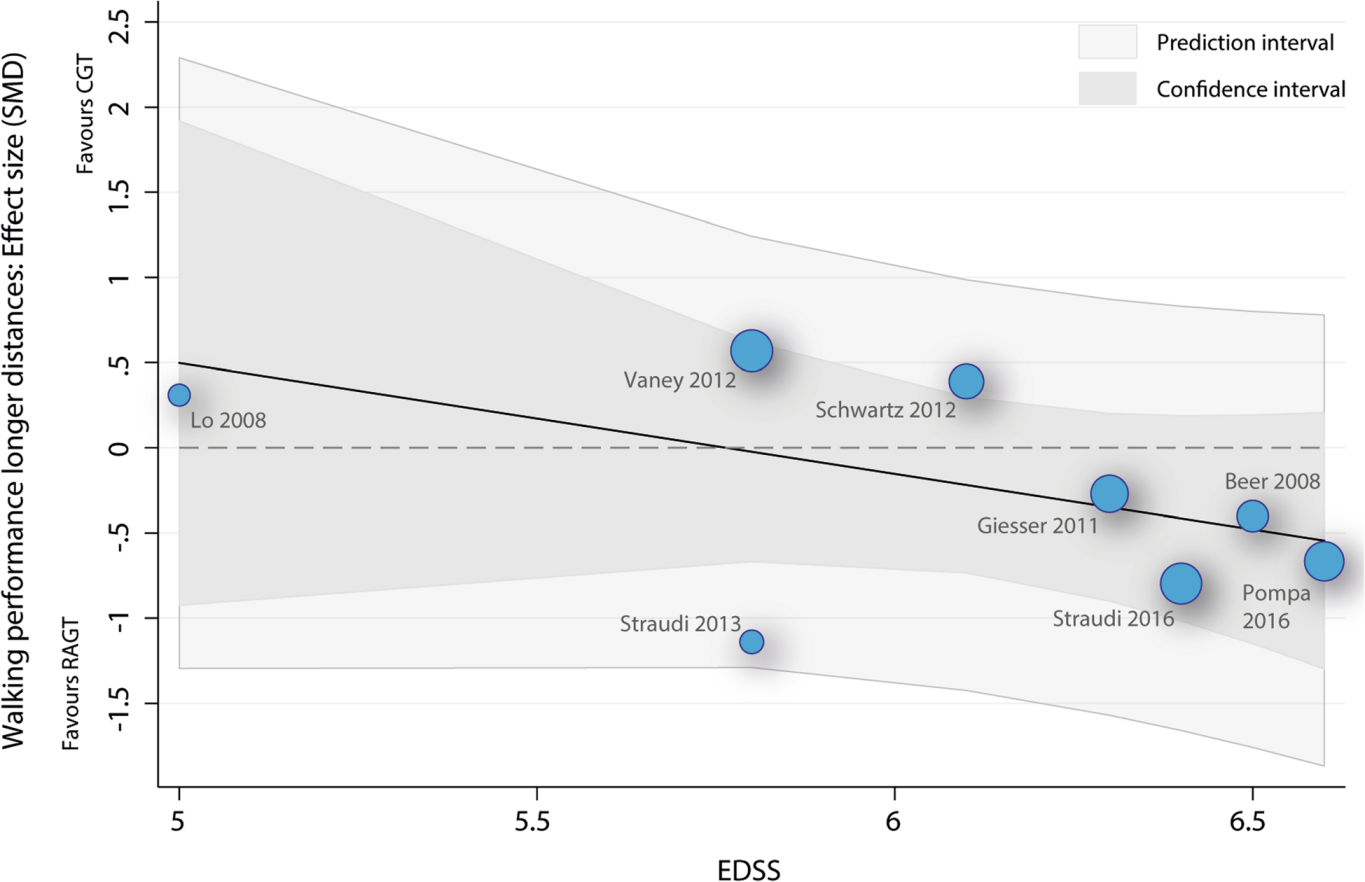
Scatterplot meta-regression walking performance over long distances. RAGT: robot-assisted gait training; CGT: conventional over-ground walking; EDSS: Expanded Disability Status Scale

The meta-regression with the EDSS score as the independent variable and walking performance on long distances as the dependent variable showed that neither the overall model (p: 0.2, r2: 0.21) or the baseline EDSS variable (b1: -0.65 (95%CI: -1.77 to 0.46), t: − 1.43, p: 0.2) were significantly related to the mean effect size (Fig. 7).

#### Risk of bias

**Fig. 8:**
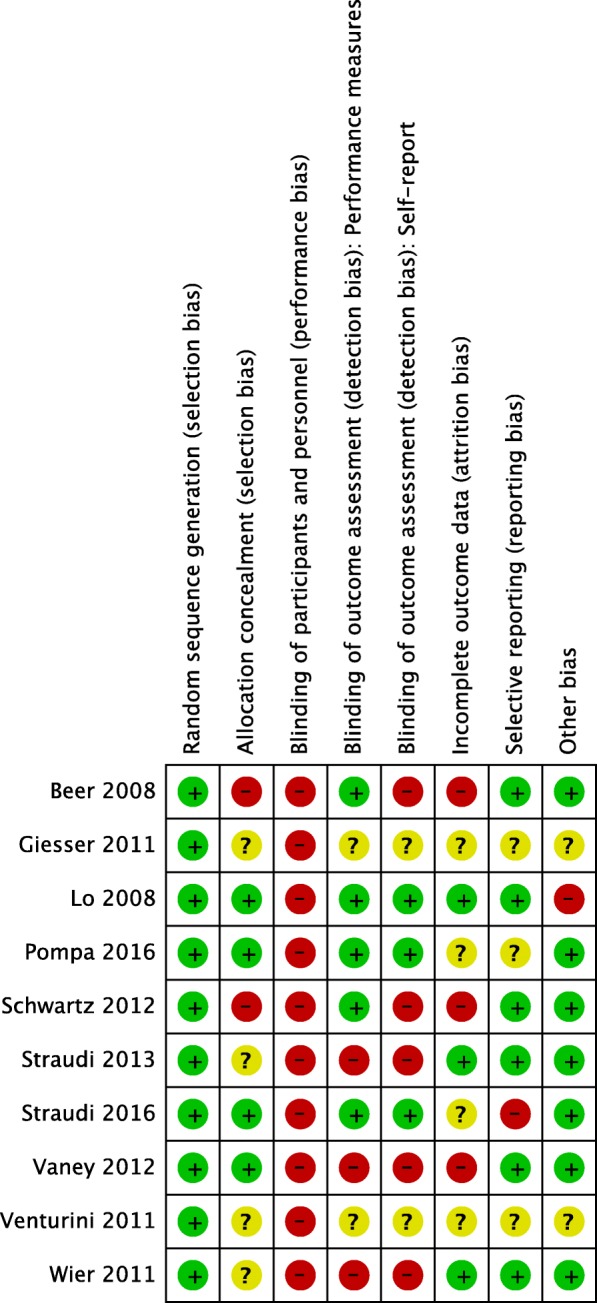
Risk of bias summary

All included studies were appraised as having a high risk of bias. This was because at least one item on the RoB assessment was evaluated as presenting a high risk of bias in each study. All studies had performed an adequate random sequence generation. In contrast only few adequately described the method of allocation concealment. All studies were classified with a high risk on the item “blinding of participants and personnel”. This was unavoidable due to the nature of the interventions. Incomplete outcome data was appraised in three studies and four studies received an unclear rating on this item. All risk of bias evaluations are presented in Fig. 8. Because there were less than ten studies included, we did not perform statistical tests for small study effects (publication bias). However, the visual inspection of the funnel plots (Additional files 2 and 3) did not show any asymmetry.

## Discussion

### Summary of main findings

Overall, this systematic review with meta-analyses and meta-regressions suggests that RAGT is not significantly more effective than CGT to train walking in people in MS. The between group differences for the walking abilities over short distances (− 0.08 SMD) and over long distances (− 0.24 SMD) were in favour of RAGT but it was not possible to reject the null-hypothesis that there is no difference between RAGT and CGT.

The data showed a possible relationship between both predictor variables (i.e. baseline walking speed and EDSS score) and the walking performance over short and long distances.

Regarding the baseline walking speed, the regression model is compatible with a moderate to large increase in effect size per 0.1 m/sec increase in baseline walking speed in favour of CGT. This was analysed for walking performance over short and long distances. However, the data are still compatible with the null-hypothesis (i.e. that baseline walking speed is not associated with the effect). Therefore, more studies are needed including participants with a wider range of walking speeds.

Similar findings were observed for the baseline EDSS values (i.e. higher disease severity was associated with a larger effect in favour of RAGT, but data were still compatible with the null-hypothesis that there is no association between disease severity (EDSS) and effect).

### Limitations

There are some limitations associated with the included studies: Information on maximal used walking speed applied during robot-assisted gait training was lacking in some studies, and the maximal possible gait speed on some of the used devices was relatively low (e.g. below 0.8 m/s). This might be one reason why conventional over-ground walking was more effective in patients with lower disabilities and faster baseline walking speeds. Another limitation of the included studies is the relatively low sample sizes and the high risk of bias of the included studies. Furthermore, the conventional gait training was only poorly described in most included studies and these interventions most probably did not form a homogenous comparison group. The lack of information did not allow to compare the CGT intervention regarding parameters such as gait speed. Furthermore, there was a lack of evidence-based criteria for progression of exercise difficulty within the CGT groups.

The limits of our systematic review and meta-regression include the low number of studies and the low variability of disabilities at baseline (i.e. most studies recruited people with an EDSS score around 6; only few reported about people with substantial less or higher disease related disabilities). Furthermore, the meta-regression is based on mean values and not on individual disability level. This could lead to aggregation bias (i.e. a study including patients with higher disabilities could show a larger effect compared to a study that included patients with less disabilities, but if we would evaluate the association between disability and effect within the studies, an inverse association could be possible). One solution to avoid aggregation bias [35] would be to perform an independent patient’s meta-analysis. Unfortunately, this was not possible due to the lack of individual data. A further limitation of our review was that we did not register the protocol in an online database such as PROSPERO. The strength of our study was the systematic approach following state of the art recommendations [36].

Another limitation might be that no studies were included comparing CGT or RAGT against a true control group (such as usual care, no treatment or waitlist), which did not receive an intervention designed to increase walking abilities. This might explain why the findings were not statistically significant, and the effect sizes were relatively small.

In addition, a moderate to large amount of heterogeneity was identified in our analyses, which might have been caused among others by several clinical variables such as i) the large difference in the design of the CGT interventions, ii) the different outcome measures, which were used to assess walking performance over short and long distances and iii) the risk of bias in included studies. Unfortunately, not enough studies were available to perform a sufficiently powered moderator analysis. This relatively high degree of heterogeneity is an important limitation of this study.

### Agreement with other studies

To our knowledge there exists one systematic review reporting about the effectiveness of robot assisted gait training in MS [37]. The authors reported that robot-assisted gait training seemed to improve several walking parameters in people with MS, such as walking speed and endurance. This is similar to the findings of our analysis, where small effect sizes in favour of robotic training were appraised on walking abilities over short and long distances. However, the authors did not perform a meta-analysis and also reported about treadmill training, which was not included in this review. Therefore, a direct comparison of the estimated effect is not possible. One previous published meta-analysis commenting about robot assisted gait training in people with MS was identified [20]. This meta-analysis appraised that conventional over-ground training showed better effects on the outcome walking speed. One critique to this meta-analysis is that it was not based on a systematic review design and only three studies were included in the analysis. Especially, no studies published after 2010 were included. Given that the majority of included studies within our review were published afterwards it is explicable why our findings deviate from the meta-analysis of Vaney et al. [19].

### Implications for practice and research

The findings of the meta-analysis indicate that none of the two interventions was superior to the other. Given the high cost of the devices and the result of the meta-regression, robot-assisted gait training should be mainly reserved for patients with higher disabilities.

The robot-assisted gait training devices are under continuous development, e.g. more degree of freedoms, virtual reality, and faster speed are incorporated. Given the similar effects of over-ground walking, these newly development models should be evaluated in controlled trials prior to their application in clinical practice. On the other side, there is a clear need for studies defining the state of the art of conventional over-ground walking training programmes. Given the huge heterogeneity within this intervention research should set out to develop guidelines, which can support clinicians during the conventional over-ground walk training. Among others clear evidence-based progression lines should be established.

## Conclusion

Overall, the weight of the available evidence suggests that robot-assisted gait training (RAGT) is not significantly more effective than conventional over-ground walking (CGT) to train walking in people in MS.

Future studies are needed to help clinicians to decide, which intervention (RAGT versus CGT) should be allocated to the individual patient. Therefore, the influence of potential moderator variables such as disease related disability or baseline walking speed should be further investigated.

## Additional files

Additional file 1: Search strategy Pubmed, the used search terms and combinations of the search terms in Pubmed (DOCX 13 kb)

Additional file 2: Funnel plot 1, the funnel plot for the outcome “short distance walking tests” (TIF 599 kb)

Additional file 3: Funnel plot 2, the funnel plot for the outcome “long distance walking tests” (TIF 745 kb)

Additional file 4: Data set RAGT CGT, the data set used for the analyses (CSV 2 kb)

## Abbreviations

CGT: Conventional over-ground walking training
EDSS: Expanded Disability Status Scale
MS: Multiple sclerosis
RAGT: Robot-assisted gait training
SMD: Standardized mean difference
