# Supplementary material for: Over-ground walking or robot-assisted gait training in people with .multiple sclerosis: does the effect depend on baseline walking speed and disease related disabilities? A systematic review and meta-regression
Source: BMC Neurol. 2019 May 8;19:93. doi: 10.1186/s12883-019-1321-7 (PMC6506946; doi:10.1186/s12883-019-1321-7)
Supplement: Supplementary file 1 — Search strategy Pubmed, the used search terms and combinations of the search terms in Pubmed (DOCX 13 kb) [file 12883_2019_1321_MOESM1_ESM.docx]

## **Additional file 1**

Search Strategy PubMed

1. **Robot**
2. **Robotic**
3. **Lokomat**
4. **#1 or #2 or #3**
5. **Gait**
6. **Walk**
7. **Walking**
8. **Speed**
9. **Timed**
10. **#5 or #6 or #7 or #8 or #9**
11. **#4 and #10**
12. **(randomized controlled trial[pt]) OR (controlled clinical trial[pt]) OR (randomized[tiab]) OR (placebo[tiab]) OR (drug therapy[sh]) OR randomly[tiab] OR trial[tiab] OR groups[tiab]) NOT (animals[mh] NOT (humans[mh] AND animals[mh]))**
13. **#11 and #12**
