# Supplementary figures and images for: Over-ground walking or robot-assisted gait training in people with .multiple sclerosis: does the effect depend on baseline walking speed and disease related disabilities? A systematic review and meta-regression
Source: BMC Neurol. 2019 May 8;19:93. doi: 10.1186/s12883-019-1321-7 (PMC6506946; doi:10.1186/s12883-019-1321-7)

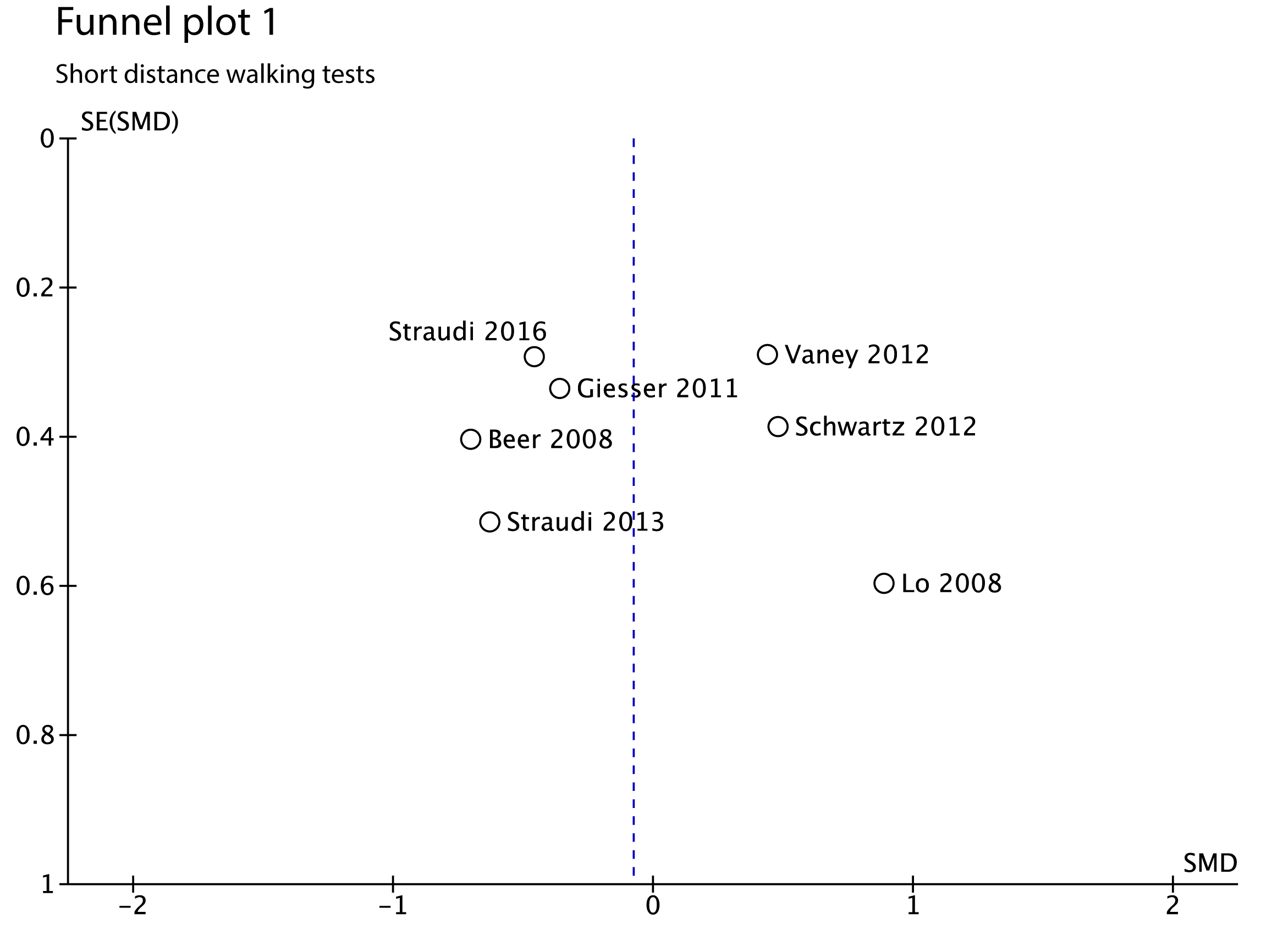

Supplement: Supplementary file 2 — Funnel plot 1, the funnel plot for the outcome “short distance walking tests” (TIF 599 kb) [file 12883_2019_1321_MOESM2_ESM.tif]

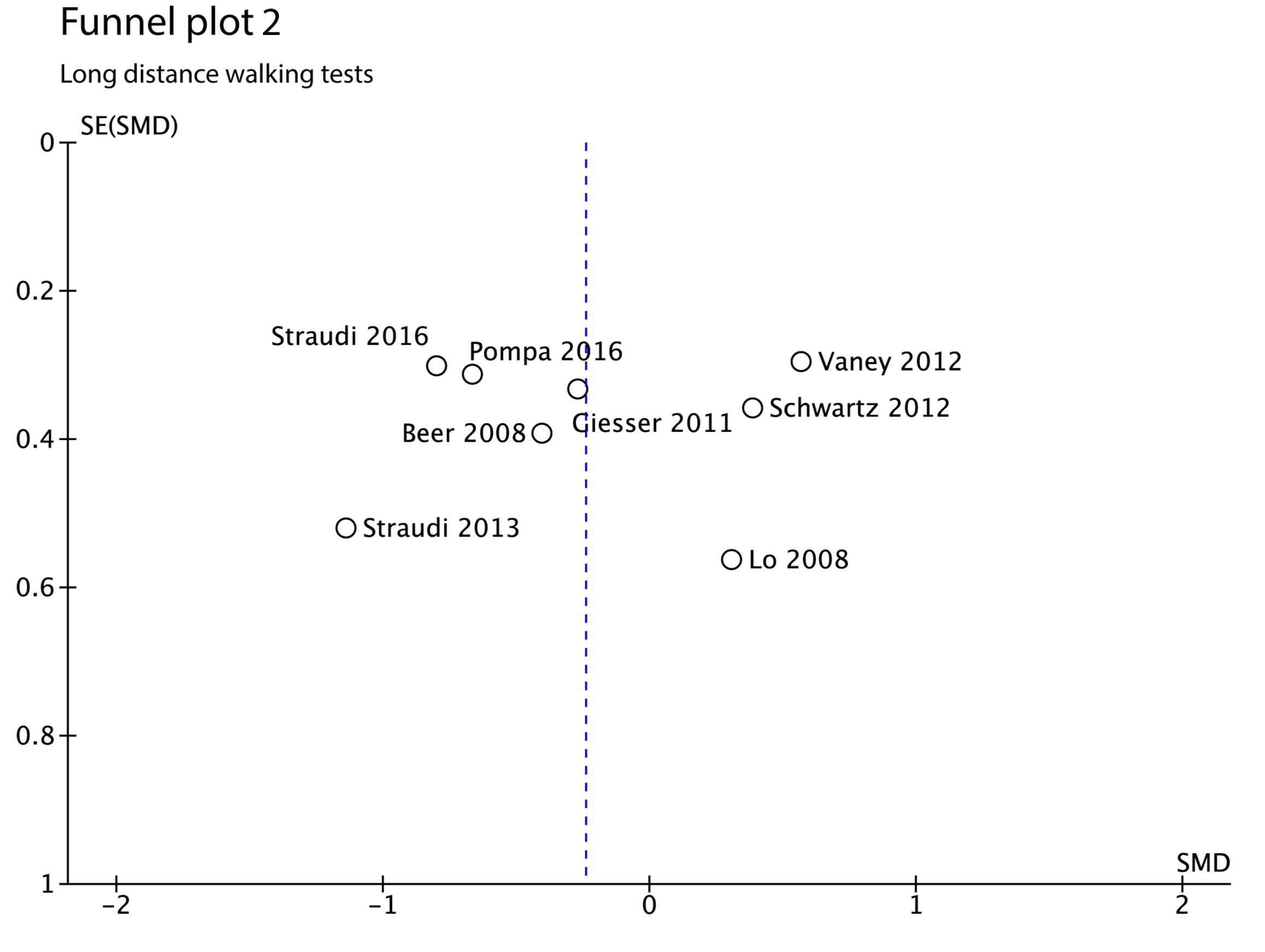

Supplement: Supplementary file 3 — Funnel plot 2, the funnel plot for the outcome “long distance walking tests” (TIF 745 kb) [file 12883_2019_1321_MOESM3_ESM.tif]
